# *Helicobacter pylori* Infection Is Associated with the Presence of Thyroid Nodules in the Euthyroid Population

**DOI:** 10.1371/journal.pone.0080042

**Published:** 2013-11-11

**Authors:** Zhe Shen, Yu’e Qin, Yi Liu, Yi Lu, Stefan Munker, Lihua Chen, Chaohui Yu, Peng Chen, Youming Li

**Affiliations:** 1 Department of Gastroenterology, the First Affiliated Hospital, College of Medicine, Zhejiang University, Hangzhou, China; 2 International Health Care Center, the First Affiliated Hospital, College of Medicine, Zhejiang University, Hangzhou, China; 3 Molecular Hepatology-Alcohol Associated Diseases, II. Medical Clinic Faculty of Medicine at Mannheim, University of Heidelberg, Mannheim, Germany; Veterans Affairs Medical Center (111D), United States of America

## Abstract

*Helicobacter pylori* infection is associated with extragastric diseases. The thyroid may be one of the targets of chronic inflammation. Here, we sought to investigate whether *H. pylori* infections were associated with the presence of thyroid nodules. A total of 988 euthyroid subjects from China were included in this cross-sectional study. Four hundred thirty-five (44.0%) subjects were diagnosed as having thyroid nodules, and 486 (49.2%) were diagnosed with *H. pylori* infections. The thyroid nodules group had a higher proportion of *H. pylori* infections than the control group (*P* = 0.002). Free thyroxine (FT4) levels were lower and the prevalence of thyroid nodules was higher in patients with *H. pylori* infection compared to those without infection, even after adjustment for age, gender, and body mass index (BMI; all *P* < 0.05). The prevalence of *H. pylori* infection showed a decreasing trend as serum FT4 level increased (*P*_trend_ = 0.020). Stepwise logistic regression analysis showed that *H. pylori* infection was significantly associated with the risk of thyroid nodules (odds ratio: 1.390, 95% confidence interval: 1.059–1.824, *P* = 0.018). Our results suggested that *H. pylori* infections were positively associated with the presence of thyroid nodules in the euthyroid population, whose thyroid functions were in the reference range.

## Introduction

*Helicobacter pylori* is a gram-negative, spiral-shaped, pathogenic bacterium that typically colonizes and infects the gastric mucosa. *H. pylori* infections and *H. pylori*-induced chronic inflammation can cause gastric diseases, such as chronic gastritis, peptic ulcers, and gastric malignancies [1,2]. Moreover, *H. pylori* infection can also cause extragastric diseases. Preliminary data have shown that there is a positive relationship between *H. pylori* infection and extragastric diseases, such as metabolic syndrome [3], nonalcoholic fatty liver disease [4], diabetes mellitus [5], and insulin resistance (IR) [6]. In addition, some reports have shown a positive correlation between *H. pylori* infections and autoimmune thyroid diseases (ATDs) [7-9]. Indeed, studies have demonstrated that some bacteria and viruses are able to mimic the antigenic profile of the thyroid cell membrane, thereby playing an important role in the onset of autoimmune diseases [10-13]. Therefore, the thyroid may be attacked by autoantibodies after *H. pylori* infection. 

Thyroid nodules are common, and benign, nodular goiters account for about 80%–90% of nodules, while thyroid cancer accounts for only about 5%–10% [14]. In the clinical setting, some patients exhibit thyroid nodules by ultrasound imaging, but have normal serum thyroid function levels. Though the vast majority of nodules are benign, the risk factors for thyroid nodules among euthyroid population have not yet been fully elucidated. Bassi et al. have demonstrated a noteworthy correlation between *H. pylori* infection and Graves’ disease, independent of hormonal status [8]. However, it is not know whether there is a link between *H. pylori* infection and the presence of thyroid nodules.

Therefore, we conducted a cross-sectional study to investigate the correlation between *H. pylori* infection and the presence of thyroid nodules in the euthyroid population.

## Materials and Methods

### Study design and subjects

From January to December 2012, we evaluated patients who underwent health screening that included thyroid ultrasounds, fasting ^13^C urea breath tests, and examination of laboratory data at the International Health Care Center, First Affiliated Hospital of Zhejiang University College of Medicine. Many company labor unions voluntarily organize for all their serving and retired staff to take part in annual health screening. All subjects voluntarily participated in this study. Individuals were excluded from this study if they had a history of thyroid diseases, including hyperthyroidism, hypothyroidism, or thyroid hormone replacement therapy for any reason or if they had thyroid dysfunction, defined as serum thyroid-stimulating hormone (TSH) greater than 4.34 mIU/L or less than 0.38 mIU/L and/or free thyroxine (free T4, FT4) greater than 24.38 pmol/L or less than 10.45 pmol/L. Additionally, all individuals who used drugs that may influence the results of ^13^C urea breath tests, such as antibiotics, histamine 2 receptor antagonists, proton pump inhibitors, or bismuths, within a 1-month period before screening, were excluded from this study. A total of 988 eligible subjects were enrolled (621 men and 367 women, with mean age of 46.88 ± 11.92 years). All subjects have not accepted eradication therapies of *H. pylori* infection in the past one year according to the history records. All procedures were approved by the Ethics Committee of Zhejiang University of College of Medicine. Each method and the potential risks were explained to the participants in detail, and all subjects gave written informed consent before the study.

### Physical examination

All subjects were required to fast overnight prior to physical examinations in the morning. After a health habit inventory was recorded, body measurement and blood pressure were measured by a trained physician. Body mass index (BMI) was then calculated as mass (kg)/height (m)^2^. Blood pressure was measured with an automated sphygmomanometer on the right arm of the followed-up individuals in a comfortable sitting position after a 5-min rest. Three measurements were taken. The second and third pressure readings were averaged, and systolic blood pressure (SBP) and diastolic blood pressure (DBP) readings were used for analysis.

### Laboratory assessments

Peripheral venous blood samples were collected after physical examination and used for the analysis of thyroid function. Serum TSH levels, FT4, and free triiodothyronine (free T3, FT3) were assessed with an ADVIA Centaur XP system (Siemens AG, Munich, Germany). The diagnosis of *H. pylori* infection was based on the result of fasting ^13^C urea breath test (^13^C-UBT), ^13^C-UBT was performed under the following conditions: patients underwent an 8-h fast, mouth washing before dosing, drinking water (100 mL) as standard meal, and subsequent administration of 75 mg ^13^C urea (Boran Pharmaceutical Co., Ltd. of Beijing, China); breath samples were then collected in two 10-mL sample plastic bags for a baseline reading and at a 30-min sampling point, with patients in a sitting position. The breath samples were analyzed by infrared heterodyne ratiometry (Huaheng Anbang Company of Beijing, China). *H. pylori* infection was considered present if the difference between the 30-min value and baseline value divided by the baseline value exceeded 4.0‰. Reference value ranges of all indexes were based on the biochemistry criteria of Department of Clinical Laboratory, The First Affiliated Hospital, College of Medicine, Zhejiang University.

### Ultrasonographic examination

Thyroid ultrasonography for all subjects was performed by a trained ultrasonographer using a Philips Ultrasound System HD11XE (Royal Dutch Philips Electronics Ltd., Amsterdam, Netherlands) with a 10-MHz linear probe. The location, size, number, shape, border, and internal echo of thyroid nodules were described in the ultrasonographic examination.

### Statistical analysis

Statistical analysis was performed with SPSS 13.0 statistical package (SPSS Inc., Chicago, IL, USA). The Kolmogorov–Smirnov test was used to assess whether continuous data were normally distributed. Continuous variables are presented as the mean and standard deviation or median and interquartile range (IQR), as appropriate. Continuous data for different groups was compared using Student’s *t*-test or the Mann–Whitney U-test. The chi-square (χ^2^) test was used for comparisons of categorical variables. Adjusted analysis was used to determine the relationships between *H. pylori* infection and thyroid function or thyroid nodules. Stepwise multiple regression analysis was used to evaluate the risk factors for thyroid nodules (backward: Wald; cutoff for entry: 0.05, for removal: 0.10). Differences with *P*-values less than 0.05 were considered statistically significant.

## Results

### Subject characteristics

Of the 988 subjects enrolled in this study, 435 (44.0%) were diagnosed with thyroid nodules, and 486 (49.2%) were diagnosed with *H. pylori* infections. Characteristics of the subjects according to thyroid nodule status are illustrated in Table 1. The average age of subjects in the thyroid nodules group was higher than that in the control group. SBP, DBP, BMI, and the presence of *H. pylori* infection were unfavorable and the proportion of women was higher in the thyroid nodules group when compared to the control group. Additionally, FT4 and FT3 levels were significantly lower in subjects with thyroid nodules than in control subjects (all *P* < 0.05). 

**Table 1:** Characteristics of study subjects according to thyroid nodule (TN) status.

Variables	Subjects with TNs (n = 435)	Subjects without TNs (n = 553)	*t*-value	*P*-value
Age (years)	50 (43–59)	43 (36–50)	9.771^a^	< 0.001
Gender (male/female, n)	240/195	381/172	19.643^b^	< 0.001
Systolic blood pressure (mmHg)	129.2 ± 18.8	124.0 ± 16.4	4.673	< 0.001
Diastolic blood pressure (mmHg)	78.1 ± 10.7	76.1 ± 10.9	2.906	0.004
Body mass index (kg/m^2^)	24.1 ± 3.0	23.5 ± 3.1	3.094	0.002
Thyroid- stimulating hormone (mIU/L)	1.67 (1.13–2.28)	1.68 (1.20–2.39)	0.689^a^	0.491
Free T4 (pmol/L)	15.70 ± 2.22	16.06 ± 2.28	2.420	0.016
Free T3 (pmol/L)	4.74 ± 0.54	4.86 ± 0.54	3.251	0.001
^13^C urea breath test (positive/negative, n)	238/197	248/305	9.483^b^	0.002

Data are expressed as mean (SD) or median (IQR). ^a^ Z value; ^b^ χ^2^ value.

### Associations between *H. pylori* infection and thyroid function or thyroid nodules

When subjects were assessed relative to their *H. pylori* infection status, the results showed that the FT4 levels were significantly lower, and the prevalence of thyroid nodules was significantly higher in subjects with *H. pylori* infection compared to control subjects, even after adjustment for age, gender and BMI (all *P* < 0.05). However, there were no differences in TSH or FT3 levels between *H. pylori*-infected subjects and controls (Table 2).

**Table 2:** Associations between *H. pylori* infection and thyroid function or thyroid nodules (TNs).

Variables	Unadjusted	Model 1	Model 2	Model 3
	Crude OR (95% CI)	aOR (95% CI)	aOR (95% CI)	aOR (95% CI)
TSH	0.966 (0.837-1.115)	0.953 (0.825-1.101)	0.977 (0.843-1.132)	0.961 (0.828-1.115)
FT4	0.916 (0.866-0.969)*	0.930 (0.878-0.985)*	0.912 (0.859-0.968)*	0.912 (0.859-0.969)*
FT3	1.158 (0.919-1.459)	1.220 (0.965-1.544)	1.153 (0.896-1.485)	1.116 (0.865-1.439)
TNs	1.486 (1.154-1.912)*	1.367 (1.049-1.780)*	1.432 (1.094-1.874)*	1.388 (1.059-1.819)*

Model 1: adjusted for age Model 2: adjusted for age and gender.

Model 3: adjusted for age, gender and body mass index.

* *P* < 0.05

Abbreviations: TSH, thyroid-stimulating hormone; FT4, free thyroxine; FT3, free triiodothyronine; OR, odds ratio; CI, confidence interval.

### Association between thyroid function and the prevalence of *H. pylori* infection

To investigate the relationship between thyroid function and the prevalence of *H. pylori* infection, all subjects were classified into average quartiles according to their TSH, FT4, and FT3 levels. For TSH, quartile 1 (Q1): TSH ≤ 1.16 mIU/L; quartile 2 (Q2): TSH 1.17–1.67 mIU/L; quartile 3 (Q3): TSH 1.68–2.34 mIU/L; quartile 4 (Q4): TSH ≥ 2.35 mIU/L. For FT4, Q1: FT4 ≤ 14.31 pmol/L; Q2: FT4 14.32–15.79 pmol/L; Q3: FT4 15.80–17.26 pmol/L; Q4: FT4 ≥ 17.27 pmol/L. For FT3, Q1: FT3 ≤ 4.45 pmol/L; Q2: FT3 4.46–4.80 pmol/L; Q3: FT3 4.81–5.11 pmol/L; Q4: FT3 ≥ 5.12 pmol/L. The prevalence of *H. pylori* infection in subjects with different quartile levels of TSH, FT4, and FT3 was analyzed.

**Figure 1:**
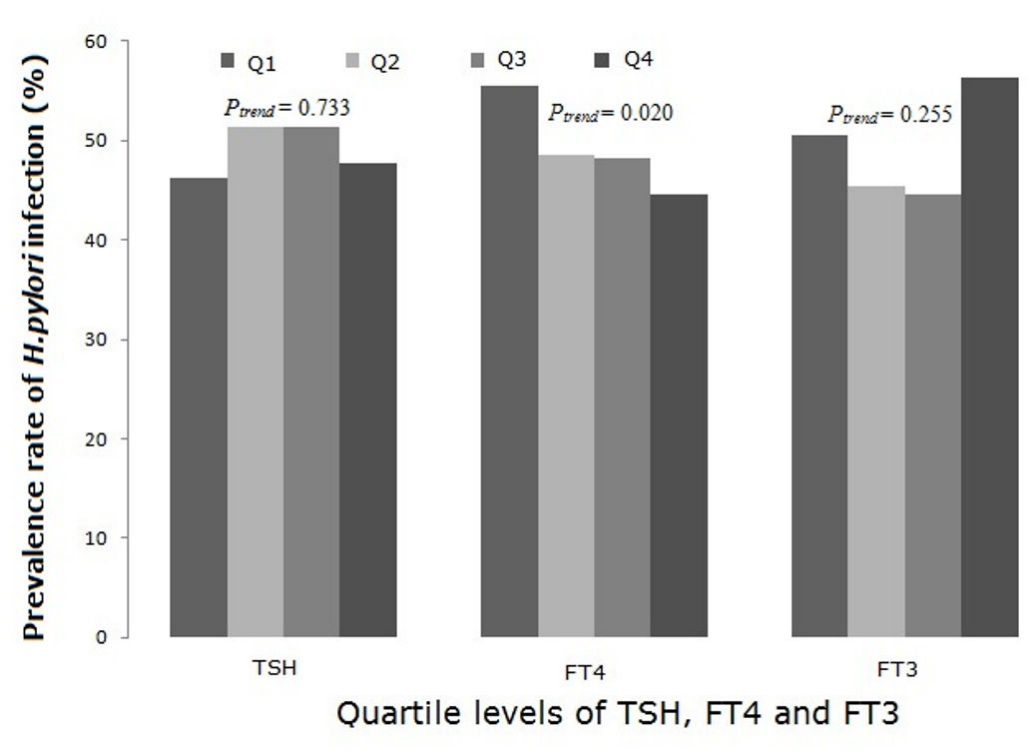
Prevalence of *Helicobacter pylori* infection according to thyroid function. Subjects were classified into different groups according to their TSH, FT4, and FT3 quartiles. The prevalence of *H. pylori* showed a decreasing trend as serum FT4 increased, while the trend was not significantly associated with serum TSH or FT3 levels. *P*-values were adjusted for age, gender and BMI status.

As shown in Figure 1, the prevalence of *H. pylori* infection showed a decreasing trend as serum FT4 levels increased. Compared to subjects with serum FT4 levels in Q1, the prevalence ratios for subjects in Q2, Q3, and Q4 were 0.88, 0.87, and 0.80, respectively. This trend was significant after adjustment for age, gender and BMI (Figure 1; *P* for trend = 0.020). However, the trends for TSH and FT3 were not statistically significant (Figure 1). These results suggested that lower FT4 levels were more likely to be related to *H. pylori* infection.

### Risk factor analysis for thyroid nodules

Univariate logistic-regression analysis was performed to evaluate the relationship between nine variables and thyroid nodules. Nine variables included age, female gender, SBP, DBP, BMI, *H. pylori* infection, TSH levels, FT4 levels, and FT3 levels. The results showed that age, female gender, SBP, DBP, BMI, FT4, FT3 and *H. pylori* infection were significantly associated with the risk for thyroid nodules (Table 3, all *P* < 0.05). Stepwise logistic regression analysis was performed to evaluate the relationship between *H. pylori* infection and thyroid nodules. The results showed that age, female gender, BMI, and *H. pylori* infection were significantly and positively associated with the risk for thyroid nodules, while TSH was inversely correlated with the risk for thyroid nodules (Table 4).

**Table 3:** Relationship between nine variables and thyroid nodules (TNs) in euthyroid population using univariate logistic-regression.

Variables	β	SE	Wald χ^2^	*P*-value	OR	95% CI of OR
Age	0.056	0.006	80.641	0.000	1.057	1.045-1.070
Female	0.588	0.133	19.474	0.000	1.800	1.386-2.337
SBP	0.017	0.004	20.966	0.000	1.017	1.010-1.025
DBP	0.017	0.006	8.327	0.004	1.017	1.006-1.029
BMI	0.064	0.021	9.372	0.002	1.067	1.023-1.111
TSH	-0.070	0.074	0.905	0.342	0.932	0.806-1.077
FT4	-0.069	0.029	5.796	0.016	0.933	0.882-0.987
FT3	-0.391	0.122	10.316	0.001	0.677	0.533-0.859
*H. pylori* infection	0.396	0.129	9.451	0.002	1.486	1.154-1.912

Abbrevations: β, partial regression coefficient; SE, standard error of partial regression coefficient; OR, odds ratio; CI, confidence interval.

**Table 4:** Risk factors for thyroid nodules (TNs) in the euthyroid population.

Variables	β	SE	Wald χ^2^	*P*-value	OR	95% CI of OR
Age	0.055	0.006	73.581	0.000	1.056	1.043–1.070
Female	0.947	0.158	35.953	0.000	2.578	1.892–3.514
BMI	0.073	0.024	8.851	0.003	1.075	1.025–1.128
TSH	-0.243	0.083	8.630	0.003	0.784	0.667–0.922
*H. pylori* infection	0.329	0.139	5.633	0.018	1.390	1.059–1.824

Abbrevations: β, partial regression coefficient; SE, standard error of partial regression coefficient; OR, odds ratio; CI, confidence interval.

## Discussion

Thyroid nodules represent a common medical problem, and the majority (> 95%) of thyroid nodules are benign [15]. Thorough histories and physical examinations, serum TSH levels, thyroid ultrasounds, and fine need aspirations (FNAs) comprise the standard evaluation of patients with thyroid nodules. Individuals with nodules demonstrating suspicious features on thyroid ultrasound in particular should undergo FNA, and/or individuals with thyroid dysfunction should undergo further examinations to identify the reason for such dysfunction; however, such individuals were not considered in this study. Thyroid nodules refer to one or more lumpy tissues representing structural abnormalities induced by various causes in thyroid tissue [16], and nodular goiters account for about 80%–90%, while thyroid cancer accounts for only about 5%–10% [14]. However, the mechanism of thyroid nodule development remains unclear. The potential for chronic infectious agents to be a causal factors for autoimmune disease has long been recognized and has recently been receiving increased attention [17,18]. Bassi et al found that there was a positive correlation between *H. pylori* infections and Grave’s disease [7,8]. Nodule formation is a type of reactive hyperplastic lesion arising from chronic inflammation. In this study, our data showed that *H. pylori* infection was significantly associated with the presence of thyroid nodules in the euthyroid population. First, the proportion of subjects with *H. pylori* infection was higher in the thyroid nodules group than in the control group. Second, the prevalence of thyroid nodules was significantly higher in subjects with *H. pylori* infection than in control subjects, even after adjustment for age, gender, and BMI. Third, logistic regression analysis further showed that *H. pylori* infection significantly contributed to the risk for thyroid nodules. Then, how can we explain the relationship between *H. pylori* infection and thyroid nodules? Molecular modeling has indicated that one type of bacteria produces a substance capable of disabling the vitamin D receptor (VDR); then, bacteria-induced VDR dysfunction could lead to low 25-D and high 1,25-D levels [19]. 1,25-D has a very high affinity for the α-thyroid receptor, and if transcription by the α-thyroid receptor is dysregulated, a cascade of metabolic dysfunction will result [20]. Further studies in animal models are needed to clarify the mechanisms mediating the relationship between *H. pylori* infection and thyroid nodules.

In this study, our data showed that FT4 levels were negatively related to *H. pylori* infection in both unadjusted and adjusted models. Additionally, our results showed that the prevalence of *H. pylori* infection decreased gradually as serum FT4 levels increased. Triantafillidis et al. assessed the relationship between *H. pylori* infection and serum thyroid hormone levels in 110 normal volunteers and found that FT4 levels were significantly different (1.04 ± 0.2 ng/dL vs. 1.17 ± 0.3 ng/dL, *P* = 0.025) in subjects who were positive and negative for *H. pylori* [21]. The results were the same as our study, but the specific mechanism was not clear.

Actually, age is an important factor influencing the prevalence of *H. pylori* infection, and age was also a significant factor for the prevalence of thyroid nodules in our study. As to describe the characteristics of the prevalence of thyroid nodules, but also to avoid selection bias of age, we assess the relationship between thyroid nodules and *H. pylori* infection by the adjusted models which are adjusted for age. Additionally, multivariate analysis found that age and female gender were significant risk factors for thyroid nodules in our study. Kim et al. reported that thyroid nodules increase with age and that their frequency is higher among women [22]. Moreover, our data showed that BMI was also significantly and positively associated with the risk for thyroid nodules, consistent with the study by Kim et al.[22] Recent studies have also shown that insulin resistance was associated with thyroid functional and morphological abnormalities [23,24]. The insulin/insulin-like growth factor-1 (IGF-1) signaling pathway has long been known to modulate thyroid gene expression and may be considered an additional important factor in thyrocyte proliferation and differentiation [25-28]. *H. pylori* infection may have a pathogenic role in the development of insulin resistance [6]. Thus, activation of the insulin pathway may be a plausible explanation for our results. Junik et al. observed the presence of negative linear correlations between thyroid volume and TSH concentration in the group of type 2 diabetics, and the authors explained that this alteration could contribute to its role in the insulin pathway [24]. According to our results, TSH was inversely correlated with the risk for thyroid nodules, and this could also be caused by the insulin pathway.

In summary, our results showed that *H. pylori* infection was positively associated with the presence of thyroid nodules in the euthyroid population. However, it is a pity that the size and number of thyroid nodules of all subjects hadn’t been provided in the study, as it is an annual health screening, a part of records on the size or number of thyroid nodules were recorded unclearly. Further studies are necessary in order to fully understand the potential mechanisms mediating this association and may help to identify the key point of disease prevention.
